# The relationship between below average cognitive ability at age 5 years and the child’s experience of school at age 9

**DOI:** 10.3389/fpubh.2025.1341797

**Published:** 2025-03-04

**Authors:** Andrea K. Bowe, Mathias Urban, Anthony Staines, Deirdre M. Murray

**Affiliations:** ^1^INFANT Research Centre, University College Cork, Cork, Ireland; ^2^School of Language, Literacy & Early Childhood Education, Dublin City University, Dublin, Ireland; ^3^School of Nursing, Psychotherapy, and Community Health, Dublin City University, Dublin, Ireland; ^4^Department of Paediatrics, Cork University Hospital, Cork, Ireland

**Keywords:** cognitive ability, school outcomes, self-concept, emotional-behavioural difficulties, public health

## Abstract

**Background:**

At age 5, while only embarking on their educational journey, substantial differences in children’s cognitive ability will already exist. The aim of this study was to examine the causal association between below average cognitive ability at age 5 years and child-reported experience of school and self-concept, and teacher-reported class engagement and emotional-behavioural function at age 9 years.

**Methods:**

This longitudinal cohort study used data from 7,392 children in the Growing Up in Ireland Infant Cohort, who had completed the Picture Similarities and Naming Vocabulary subtests of the British Abilities Scales at age 5. Principal components analysis was used to produce a composite general cognitive ability score for each child. Children with a general cognitive ability score more than 1 standard deviation (SD) below the mean at age 5 were categorised as ‘Below Average Cognitive Ability’ (BACA), and those scoring above this as ‘Typical Cognitive Development’ (TCD). The outcomes of interest, measured at age 9, were child-reported experience of school, child’s self-concept, teacher-reported class engagement, and teacher-reported emotional behavioural function. Binary and multinomial logistic regression models were used to examine the association between BACA and these outcomes.

**Results:**

Compared to those with TCD, those with BACA had significantly higher odds of never liking school [Adjusted odds ratio (AOR) 1.82, 95% CI 1.37–2.43, *p* < 0.001], of being picked on (AOR 1.27, 95% CI 1.09–1.48) and of picking on others (AOR 1.53, 95% CI 1.27–1.84). They had significantly higher odds of experiencing low self-concept (AOR 1.20, 95% CI 1.02–1.42) and emotional-behavioural difficulties (AOR 1.34, 95% CI 1.10–1.63, *p* = 0.003). Compared to those with TCD, children with BACA had significantly higher odds of hardly ever or never being interested, motivated and excited to learn (AOR 2.29, 95% CI 1.70–3.10).

**Conclusion:**

Children with BACA at school-entry had significantly higher odds of reporting a negative school experience and low self-concept at age 9. They had significantly higher odds of having teacher-reported poor class engagement and problematic emotional-behavioural function at age 9. The findings of this study suggest BACA has a causal role in these adverse outcomes. Early childhood policy and intervention design should be cognisant of the important role of cognitive ability in school and childhood outcomes.

## Introduction

An important early milestone, for both children and parents, is the transition into primary school, which typically occurs around 5 years of age for children in Ireland (1, 2). The early experience of school lays the foundation for future academic and social development (3). While only embarking on their educational journey, substantial inequalities in cognitive ability will already exist (4) Around 2% will have a standardised cognitive ability score more than two standard deviations (SDs) below the mean and may meet criteria for an intellectual developmental disorder (IDD) (5). A much larger proportion, approximately 14%, will have cognitive ability that lies between one and two SDs below the mean (6, 7).

Large epidemiological studies have demonstrated that cognitive ability in childhood contributes to important adult outcomes including educational attainment, social mobility, cardiovascular disease, and mental health (8–11). Far less has been published on how cognitive ability affects early childhood experiences and outcomes, a formative one being a child’s early experience of school. A small cohort study by McIntyre et al. found children with an IDD had significantly more teacher-reported problem behaviour, fewer self-regulation and social skills, and poorer student-teacher relationships (1). Children with below average cognitive ability, not meeting criteria for an IDD, were excluded from their study. The early school experience for these children, whose difficulties may be more likely to go unrecognised and unsupported, has not previously been investigated.

In the early years of education, children with below average cognitive ability (BACA) may not display overt signs of difficulty. However, as the cognitive complexity of academic tasks increases, the child may struggle to keep up with their peers and experience academic failure which may in turn influence, among other things, their experience of school, their self-concept, their engagement with learning, and their emotional and behaviour function (12).

A child’s self-concept refers to the child’s personal perception of their self and is thought to play a critical role in how a child functions in almost all aspects of life, including how they cope with challenges (13–15). It is generally viewed as a hierarchical construct, with “general self-concept,” one’s over-arching view of self, being comprised of multiple, correlated, domain-specific self-concepts, for example academic self-concept and physical self-concept (14). Given the importance of self-concept for the adaptive functioning of the child it is essential factors which may contribute to negative self-concept are explored and understood.

Emotional functioning broadly describes the experience, expression, and regulation of both positive and negative emotions and is intimately intertwined with a child’s behavioural function, one’s actions and reactions to their environment (16, 17). Problematic emotional-behavioural function in childhood is common, with an estimated prevalence between 6 and 14% (18–21). If persistent, frequent, distressing, and impacting function in other domains of life, it may constitute an emotional-behavioural disorder (18, 19). Common disorders in childhood include depression, anxiety, attention deficit hyperactivity disorder and conduct disorder (18, 19). Screening tools, of which the Strengths and Difficulties Questionnaire is one, can be completed by parents, the child, or teachers and aim to detect problematic emotional-behavioural functioning (22).

Early subtle signs of academic difficulty in the early school years may go unnoticed or may be attributed to other causes such as personality or behavioural problems. Compulsory standardised testing in the Irish school system occurs when a child is in 2nd class (approximately 8 years of age), and until this point children with BACA may struggle unrecognised and often unsupported (23). The Growing Up in Ireland (GUI) Infant ‘08 Cohort study, which directly measured cognitive ability at age 5 and interviewed both children and their teachers at age 9 provides an opportunity to explore the relationship between BACA and a child’s experience of school, their self-concept, their classroom engagement, and their emotional-behavioural function. If the earliest years of formal education are adversely affected by cognitive deficits pre-dating school entry, this provides further rationale and impetus for very early intervention, prior to starting school, for at risk children.

The relationships between BACA and the aforementioned outcomes have multiple important confounders which must be addressed if a causal association between BACA and each outcome is to be established. Published literature was carefully examined to determine the appropriate confounding variables for inclusion and directed acyclic graphs were used to document our assumptions about the relationships between the potential cause (BACA), outcomes, and confounders (24). Among the most important confounding factors is the sociodemographic background of the child, including their gender, socioeconomic position, and cultural background, which have been shown to be strongly associated with cognitive ability in childhood and the outcomes of interest (25–31).

Child factors including the gestational age at which the child was born and the child’s individual temperament have also been shown to be associated with both cognitive ability and self-concept, emotional-behavioural function, and the formation of student teacher relationships (15, 32–39). The relationship between BMI and cognitive ability in childhood has not been clearly established in the literature with some studies suggesting the association is completely mediated by socioeconomic factors, while others suggest BMI itself has a causal relationship with cognitive ability (40, 41). However, the respective associations between high BMI and low self-concept and problematic emotional behavioural function in childhood are well documented (42–44).

Family factors such as the structure of the family, the parent–child relationship, and maternal mental health have been shown to be associated with both cognitive ability and self-concept, learning engagement, and emotional-behavioural function (45–51). Multiple studies have shown that family size has an inverse association with childhood IQ, and many posit the ‘resource dilution’ model as an explanation, whereby the family resources including parental time and income reduce as family size increases (45). There is consistent evidence that children in single parent households are at increased risk of lower academic achievement, lower self-concept and problematic emotional-behavioural function compared to those in two-parent households, with similar effects documented for the effect of maternal depression (46, 47, 50).

The aim of this study was to examine the causal association between below average cognitive ability at age 5 and child-reported experience of school and self-concept, and teacher-reported class engagement and emotional-behavioural function at age 9. Our hypothesis was that early below average cognitive ability has an adverse relationship with self-concept, engagement with learning, and emotional-behavioural function, as children with below average cognitive ability may struggle, often unrecognised, to cope with increasing academic demands.

## Methods

### Study design

This study is a secondary analysis of data collected in the GUI Infant Cohort, a nationally-representative longitudinal study of infants in Ireland, which contains information collected from primary caregivers (PCGs), teachers, and the children themselves (52, 53). The Strengthening the Reporting of Observational Studies in Epidemiology (STROBE) guidelines were followed in the conduct and reporting of this study (54). Ethical approval for the GUI Infant Cohort was granted by a dedicated Research Ethics Committee convened by the Department of Children and Youth Affairs. Written consent was obtained from all participants in the study. PCGs were asked to provide consent to contact the child’s school. Where a child had not yet started school at the time of the 5 year questionnaire, PCGs were asked for details of their intended school, and if this had not been decided they were recontacted prior to the start of school term. Secondary analysis of the GUI dataset does not require additional ethical approval (52, 53).

### Study population

Families with infants born between 1st December 2007 and 30th June 2008 (approximately *n* = 41,000 births) were identified from the Child Benefit Register, a register used to administer universal child benefit payments in Ireland, with the aim of carrying out the first wave of data collection between September 2008 and March/April 2009 when the infant would be 9 months old. Potential participants were stratified according to marital status of claimant, county of residence, nationality, and number of children in the claim, before a systematic selection procedure with a random start and constant sampling fraction were applied. Full details of the GUI study are available (55). Wave 1 recruited 11,134 nine-month-old infants, representing a 65% response rate. These 11,134 infants made up the target sample for subsequent waves (excluding those who moved outside of Ireland, who definitively opted out of the study, or who passed away during study period). A further three waves of in-person data collection occurred at age 3 (*n* = 9,793 participants), age 5 (*n* = 9,001), and age 9 years (*n* = 8,032) (56). Included in this study are children (*n* = 7,392) who completed standardised cognitive assessments at age 5 years and follow-up at age 9. Children with trisomy 21 (*n* = 29) and cerebral palsy (*n* = 11) were excluded.

### Exposure

The exposure of interest was below average cognitive ability (BACA) at age 5. This was directly assessed using two core subtests of the British Ability Scales (BAS) Early Years Battery 2nd Edition, administered in the child’s home by a trained interviewer (57). The BAS has demonstrated construct validity as a measure of cognitive ability and has high reliability (58).

The 36-item Naming Vocabulary subtest measured verbal ability in the English language. The child was requested to name everyday items, of increasing difficulty, displayed from a picture book. The Naming Vocabulary subtest primarily measures the broad ability of comprehension—knowledge or crystallized intelligence (Gc). The narrow abilities tested are language development, which refers to the ability to understand and communicate using language, and lexical knowledge, which refers to the knowledge of words and their meanings (59, 60). The 33-item Picture Similarities subtest consisted of the child being shown four pictures and requested to match a fifth, based on a shared characteristic or construct. The Picture Similarities subtest primarily measures fluid intelligence (Gf). It tests the narrow ability of ‘induction’, which is the ability to discover underlying principles or rules governing a problem (59, 60). A standardised score for each scale, adjusted for both item difficulty and age (within a 3 month age band), was calculated (61).

Multiple BAS subtest scores can be summed to produce a General Conceptual Ability Score. Due to time constraints, only two core subtests were administered in the GUI study which means this could not be done. To produce a composite score Principal Components Analysis (PCA) was used, as in previous research (62). PCA of the two BAS subtests confirmed the presence of a general underlying cognitive ability factor. Principal component 1 (PC1) accounted for 64% of the total variance among the subtests. The Pearson correlation between this factor and the observed variable was 0.80 for Picture Similarities and 0.80 for Naming Vocabulary. PC1 was standardised to produce a general cognitive ability (GCA) score with a mean of 100 and a SD of 15. Children with a GCA score < 85 (more than 1 SD below the mean) were categorised as ‘Below Average Cognitive Ability’ (BACA) and those scoring ≥85 were categorised as typical cognitive development (TCD). Children with a GCA score < 70 (more than 2 SD below the mean) were included in the BACA group in the main analyses. In supplementary analyses (Supplementary Tables S2–S7) the BACA group was split into those with a GCA score < 70 and those with a score between 70 and 85 (1–2 SD below the mean).

### Outcomes

There were four outcomes of interest, all of which were measured at age 9.

1. the child-reported experience of school.
2. the child’s self-concept.
3. the teacher-reported class engagement.
4. the teacher-reported emotional behavioural function of the child.

### Child-reported outcomes

The child-reported school experience was measured using the child’s response to a number of questionnaire items, assessing whether the child liked school (“always like it,” “sometimes like it,” “never like it”), how well they thought they were doing in their school work (“well,” “average/ok,” “poorly”), whether they thought over the last year anyone (child or adult) had picked on them (“yes,” “no”), or whether they thought over the last year they had picked on anyone else (“yes,”“no”).

The child’s self-concept was measured using a modified version of the Piers-Harris Self-Concept Scale 2nd Edition, consisting of 32 items with a yes/no response (63). This provided a measure of overall self-concept, as well as self-concept across six subscales of intellectual and school status, behavioural adjustment, physical appearance and attributes, freedom from anxiety, popularity, and happiness and satisfaction (63). Accepted cut-offs are provided by the scale authors to categorize scores. For the purpose of this study these categories were collapsed into two categories—‘Low’ which contained all those categorised as very low, low, or low average, and ‘Average or High’ which contained all those categorised as average, high average, or high.

### Teacher-reported outcomes

Teachers were asked to complete a postal questionnaire when the child was aged 5 and 9. At both age 5 and age 9, the teacher was asked about learning limitations and the provision of additional resources. At age 9 the child’s engagement in the classroom was investigated using teacher-reported responses (“always/almost always,” “sometimes,” “hardly ever/never”) to a number of questions regarding the child’s usual interest, behaviour, and involvement in class activities and learning. For example, “Would you say the study child is confident to try new activities, initiate ideas, and to speak in a familiar group?”

The teacher-reported emotional-behavioural function of the child was measured using the Strengths and Difficulties Questionnaire (SDQ), which has been shown to be a reliable and valid instrument (64). The SDQ asks the teacher to respond, using a 3-point Likert where 1 = not true, 2 = somewhat true, and 3 = certainly true, to 25 statements about the child’s behaviour over the last 6 months or school year. These 25 items make up 5 subscales of 5 items each—Emotional subscale (Example item—“Often unhappy, downhearted or tearful”), Conduct subscale (Example item. “Often has temper tantrums or hot tempers”), Hyperactivity subscale (Example item “Constantly fidgeting or squirming”), Peer problems subscale (Example item “Often fights with other children or bullies them”), and Pro-social subscales (Example item—“Considerate of other peoples feelings”). The questionnaire provides a total difficulties score, with a higher score indicating more difficulty, along with scores for each subscale. In this study children with scores in the ‘slightly raised’, ‘high’, or ‘very high’ range were categorised as ‘High’ and children scoring below this as ‘Average’ (65). Full details of the study outcomes are described in a data dictionary contained in Supplementary Table S1.

### Covariates

The existing literature and directed acyclic graphs, created using ‘DAGitty’ (24), were used to carefully choose relevant model-specific covariates for each outcome (Supplementary Figures S1–S4). All covariates were measured when the child was aged 5 years, unless otherwise specified. Sociodemographic characteristics included child gender (male/female), siblings (yes/no), partner living in the household (yes/no), PCG highest education (lower secondary or less, secondary, technical or vocational, certificate or diploma, primary degree, post degree qualification), household social class (professional worker, managerial and technical, non-manual, skilled manual, semi-skilled, unskilled, all others gainfully occupied and unknown, validly no social class), and income quintile (1 lowest–5 highest). The child’s body mass index (BMI) was derived from the child’s height and weight directly measured by the interviewer.

The parent–child relationship was measured using the 15-item Pianta Child Parent Relationship Scale (CPRS), which demonstrated acceptable reliability and validity in the pilot phase of the study (61). The primary caregiver was asked to indicate the current applicability of 15 statements to their relationship with the child using a five-point Likert scale, where 1 = definitely does not apply and 5 = definitely applies. Seven items related to getting on with the child, for example– “I share an affectionate, warm relationship with my child,” formed a ‘positive aspects’ subscale. Scores could range from 7 to 35 with a higher score indicating a more positive relationship. Eight items related to the caregiver’s perception of difficulties in the relationship, for example—“My child and I always seem to be struggling with each other” formed the ‘conflicts’ subscale. Scores could range from 8 to 40 with a higher score indicating more difficulties (57, 61).

Child temperament was measured using 12 adapted items from the parent-reported Short Temperament Scale for Children (STSC), which demonstrated reliability and validity in both the pilot phase of the study and in previous literature (57, 66). The parent was asked to indicate the answer that best described the current behaviour of the child using a six-point Likert scale where 1 = almost never and 6 = almost always. Three subscale scores were produced—Persistence (4 items, example item—“When this child starts a project such as a puzzle or model, he/she works on it without stopping until it is completed, even if it takes a long time”), reactivity [4 items, example item—“When shopping together, if I do not buy what this child wants (e.g., sweets, clothing), he/she cries and yells”], and sociability [4 items (reverse coded), example item—“This child is shy with strange adults”].

At the 9-month survey, the gestational age of the infant, the PCG cultural background (Irish, other white background, African or other black background, Chinese or other Asian background, Other), and PCG mental health were measured, the latter using The Centre for Epidemiological Studies Depression Scale (CES-D), a validated screening tool for depression in the general population (67). All covariates are described in Supplementary Table S1.

### Statistical analysis

Analyses were conducted using SPSS software Version 28. A weighting variable, calculated by the GUI study team using a minimum information loss algorithm, to adjust for non-response and attrition was applied to all analyses (56). Missing data were managed using complete case analysis. The characteristics of the population were described using counts, percentages, and chi-square tests for categorical data; means, SDs, and t-tests for continuous normally distributed data; medians, interquartile ranges (IQRs), and Mann–Whitney U tests for continuous non-normally distributed data. Binary and multinomial logistic regression models were used to examine the association between BACA and the outcomes of interest. Models were adjusted using the DAG implied adjustment set for the estimand of interest. Unadjusted and adjusted odds ratios (AOR) with 95% confidence intervals (CI) were reported.

## Results

### Characteristics of study population

The characteristics of the 7,392 children included in the study are shown in Table 1. Children with BACA at age 5 (*n* = 1,106, 15.0%) are compared to those without across key characteristics. There were significant differences between the groups across all socioeconomic variables. Compared to the TCD group, a higher proportion of PCGs of the BACA group had second level or lower education (40.0% vs. 27.8%), were in the lowest two income quintiles (60.3% vs. 38.5%), were in single parent households (21.2% vs. 14.9%), and a lower proportion were in social classes 1 or 2 (26.4% vs. 48.0%). Among those with BACA, 44.9% (461/1,027) were reported by the teacher as having a limitation to the amount or kind of activity they could undertake in school at age 5 and 28.1% (289/1,027) were provided with additional support or resources (Supplementary Table S3). Among those with a GCA score < 70, 30% were reported by their teacher as having no limitation in the type or amount of school activities they could undertake. Of those with a recognised limitation, 21% were not provided with additional help or resources. Among those with a GCA score between 70 and 85, 41% were reported to have a limitation in the amount or kind of school activity they could undertake, of whom 42% were not provided with special help or resources.

**Table 1:** Characteristics of study population and comparison of characteristics between those who did and did not have below average cognitive ability at age 5 years.

	Valid	Total *n* = 7,392	Typical cognitive development *n* = 6,286	Below average cognitive ability *n* = 1,106	*p*-value
Child characteristics					
Gender	7,392				
Male		3,783 (51.2)	3,131 (49.8)	652 (59.0)	<0.001^a^
Gestational age	7,375				
N, mean (sd^b^)		39.5 (2.1)	6,277, 39.5 (2.1)	1,097, 39.5 (2.1)	0.802^c^
Child temperament					
Sociability—n, median (IQR^d^)	7,389	4.0 (3.0–5.0)	6,285, 4.0 (3.0–5.0)	1,104, 3.8 (2.8–4.8)	0.014^e^
Persistence—n, median (IQR^d^)	7,373	4.3 (3.5–5.0)	6,279, 4.3 (3.5–5.0)	1,095, 4.0 (3.0–4.8)	<0.001^e^
Reactivity—n, median (IQR^d^)	7,381	2.3 (1.8–3.0)	6,282, 2.3 (1.8–3.0)	1,098, 2.3 (1.8–3.3)	<0.001^e^
Body mass index—kg/m^2^	7,319	16.3 (1.7)	6,231, 16.3 (1.7)	1,088, 16.3 (1.7)	0.179^c^
General cognitive ability score	7,392				
N, mean (sd^d^)		99.8 (14.9)	6,286, 103.8 (12.1)	1,105, 77.2 (6.7)	<0.001^c^
Sociodemographic characteristics					
Household location					
Urban	7,370	3,062 (41.5)	2,588 (41.2)	474 (43.3)	
Rural		4,308 (58.5)	3,688 (58.8)	620 (56.7)	0.207^a^
Partner in household	7,392				
Yes		6,224 (84.2)	5,352 (85.1)	872 (78.8)	<0.001^a^
Siblings	7,391				
Yes		6,479 (87.7)	5,514 (87.7)	965 (87.3)	0.755^a^
Cultural background	7,375				
Irish		6,398 (86.8)	5,598 (89.1)	800 (73.3)	
Other white		642 (8.7)	457 (7.3)	185 (17.0)	
African/Black		156 (2.1)	93 (1.5)	63 (5.8)	
Chinese/Asian		151 (2.0)	116 (1.8)	35 (3.2)	
Other		28 (0.4)	20 (0.3)	8 (0.7)	<0.001^a^
PCG Highest education	7,391				
Lower secondary or less		965 (13.1)	722 (11.5)	243 (22.0)	
Secondary		1,227 (16.6)	1,027 (16.3)	200 (18.0)	
Technical or vocational		1,430 (19.3)	1,169 (18.6)	261 (23.6)	
Certificate or diploma		1,605 (21.7)	1,421 (22.6)	184 (16.7)	
Primary degree		851 (11.5)	746 (11.9)	105 (9.5)	
Post degree qualification		1,313 (17.8)	1,201 (19.1)	112 (10.1)	<0.001^a^
Household social class	7,392				
Professional workers		934 (12.6)	858 (13.6)	76 (6.9)	
Managerial and technical		2,380 (32.2)	2,164 (34.4)	216 (19.5)	
Non-manual		1,334 (18.0)	1,126 (17.9)	208 (18.8)	
Skilled manual		1,115 (15.1)	868 (13.8)	247 (22.3)	
Semi-skilled		668 (9.0)	525 (8.4)	143 (12.9)	
Unskilled		110 (1.5)	95 (1.5)	15 (1.4)	
All others gainfully occupied		76 (1.0)	51 (0.8)	25 (2.3)	
Validly no social class^f^		775 (10.5)	599 (9.5)	176 (15.9)	<0.001^a^
Equivalised Income Quintile^g^	7,009				
Lowest		1,448 (20.7)	1,107 (18.5)	341 (33.3)	
2		1,472 (21.0)	1,194 (20.0)	278 (27.0)	
3		1,383 (19.7)	1,207 (20.2)	176 (17.1)	
4		1,360 (19.4)	1,231 (20.6)	129 (12.5)	
Highest		1,346 (19.2)	1,240 (20.7)	106 (10.3)	<0.001^a^
PCG depression score	7,275				
N, median (IQR)		1.0 (0.0–3.0)	6,223, 1.0 (0.0–3.0)	1,052, 1.0 (0.0–4.0)	0.052^e^
Relationship characteristics					
Parent–child relationship					
Positive aspects^h^^—^n, median (IQR)	7,380	35.0 (33.0–35.0)	6,285, 35.0 (33.0–35.0)	1,095, 34.0 (33.0–35.0)	<0.001^e^
Conflict^i^—n, median (IQR)	7,384	14.0 (10.0–19.0)	6,284, 14.0 (10.0–18.0)	1,100, 15.0 (11.0–20.0)	<0.001^e^

a Pearson’s Chi-squared test.

b sd, standard deviation.

c Independent samples t-test.

d Interquartile range.

e Independent samples Mann–Whitney U Test.

f Validly no social class consists of households where both caregivers are currently economically inactive and have not held any previous employment in the past.

g Equivalised Income Quintile is based on the disposable household income (total gross income less statutory deductions), divided by the equivalised household size which takes account of differences in size and composition of households.

h Higher score indicates more positive relationship.

i Higher score indicates more conflict.

### Child-reported experience of school at age 9 years

Overall, 94.8% of children reported they always or sometimes liked school at age 9 (Supplementary Table S8). After adjusting for relevant confounding variables (Supplementary Figure S1), compared to those in the TCD group the odds of never liking school were 1.8 times higher [111 (10.2%) vs. 290 (4.7%), AOR 1.82, 95% CI 1.37–2.43, *p* < 0.001] for children with BACA. Children with BACA had significantly higher odds of being picked on [473 (46.8%) vs. 2,394 (40.1%), AOR 1.27, 95% CI 1.09–1.48] and of picking on others [231 (23.0%) vs. 822 (14.0%), AOR 1.53, 95% CI 1.27–1.84].

### Child’s self-concept

After appropriate adjustment for confounding (Supplementary Figure S2), children with BACA at age 5 had significantly higher odds of experiencing low overall self-concept at age 9 (AOR 1.20, 95% CI 1.02–1.42, *p* = 0.028; Table 2). The odds of experiencing low intellectual self-concept were 1.4 times higher (AOR 1.38, 95% CI 1.17–1.63, *p* < 0.001), a measure which reflects both their assessment of intellectual ability and school performance, but also their future expectations about achievement. In both groups a substantial proportion of children reported low self-concept in happiness and satisfaction (TCD 32.0% vs. BACA 43.2%, *p* < 0.001), but for children in the BACA, the odds were still 1.3 times higher than those in the TCD group (AOR 1.29, 95% CI 1.10–1.50, *p* < 0.001).

**Table 2:** Child’s self-concept at age 9 years.

	Valid	Total	Typical cognitive development	Below average cognitive ability	*p*-value	Odds ratio (95% CI^b^)	Adjusted odds ratio^c^ (95% CI)	*p*-value
Overall self-concept	6,702							
Average or high		4,860 (72.5)	4,236 (73.8)	624 (64.7)		Ref		
Low		1,842 (27.5)	1,501 (26.2)	341 (35.3)	<0.001^a^	1.54 (1.34–1.78)	1.20 (1.02–1.42)	0.028
Intellectual and school status	6,671							
Average or high		4,960 (74.4)	4,335 (75.9)	625 (65.2)		Ref		
Low		1,711 (25.6)	1,378 (24.1)	333 (34.8)	<0.001^a^	1.68 (1.45–1.94)	1.38 (1.17–1.63)	<0.001
Behavioural adjustment	6,845							
Average^d^		4,388 (64.1)	3,841 (65.6)	547 (55.5)		Ref		
Low		2,457 (35.9)	2,018 (34.4)	439 (44.5)	<0.001^a^	1.53 (1.33–1.75)	1.27 (1.09–1.48)	0.003
Physical appearance and attributes	6,799							
Average^d^		5,155 (75.8)	4,449 (76.6)	706 (71.3)		Ref		
Low		1,644 (24.2)	1,360 (23.4)	284 (28.7)	<0.001^a^	1.32 (1.13–1.53)	1.16 (0.98–1.38)	0.081
Freedom from anxiety	6,878							
Average or high		5,317 (77.3)	4,584 (77.9)	733 (73.5)		Ref		
Low		1,561 (22.7)	1,297 (22.1)	264 (26.5)	0.002^a^	1.27 (1.09–1.49)	1.03 (0.86–1.22)	0.781
Popularity	6,810							
Average or high		5,214 (76.6)	4,524 (77.7)	690 (69.8)		Ref		
Low		1,596 (23.4)	1,298 (22.3)	298 (30.2)	<0.001^a^	1.51 (1.30–1.75)	1.21 (1.02–1.44)	0.028
Happiness and satisfaction	6,877							
Average^d^		4,562 (66.3)	3,997 (68.0)	565 (56.8)		Ref		
Low		2,315 (33.7)	1,885 (32.0)	430 (43.2)	<0.001^a^	1.62 (1.41–1.85)	1.29 (1.10–1.50)	0.001

a Pearson’s Chi-squared test.

b CI, Confidence Interval.

c Adjusted for child gender, gestational age, siblings, primary caregiver education, household income, parent–child relationship, child BMI, cultural background, child temperament, partner in household, maternal mental health.

d Where ‘Average’ is listed alone no child score in the ‘high average’ or ‘high’ categories.

### Teacher-reported class engagement at age 9 years

As shown in Table 3, after adjustment for confounding (Supplementary Figure S3) children with BACA had less positive class engagement compared to their peers with TCD. They had significantly higher odds of hardly ever or never being interested, motivated and excited to learn (10.1% vs. 4.3%, AOR 2.29, 95% CI 1.70–3.10, *p* < 0.001), of hardly ever or never being confident to try new activities, initiate ideas, and speak in a familiar group (16.0% vs. 6.0%, AOR 2.73, 95% CI 2.12–3.50, *p* < 0.001), of hardly ever or never maintaining attention and concentrate (13.3% vs. 6.2%, AOR 2.42, 95% CI 1.85–3.16, *p* < 0.001,) and of hardly ever or never sustaining involvement and persevering particularly when trying to solve a problem (19.5% vs. 7.9%, AOR 2.75, 95% CI 2.17–3.50, *p* < 0.001).

**Table 3:** Teacher-reported classroom engagement and behaviour at age 9 years.

	Valid	Total	Typical cognitive development	Below average cognitive ability	*p*-value	Odds ratio (95% CI^b^)	Adjusted odds ratio^c^ (95% CI)	*p*-value
Shows an interest in classroom activities	6,729							
Always/almost always		5,175 (76.9)	4,542 (79.1)	633 (64.1)		Ref		
Sometimes		1,406 (20.9)	1,094 (19.1)	312 (31.6)		2.05 (1.76–2.38)	1.49 (1.25–1.78)	<0.001
Hardly ever/never		148 (2.2)	105 (1.8)	43 (4.4)	<0.001	2.90 (2.01–4.18)	2.01 (1.31–3.09)	0.001
High level of involvement in self-chosen activities	6,731							
Always/almost always		5,071 (75.3)	4,468 (77.8)	603 (61.2)		Ref		
Sometimes		1,476 (21.9)	1,159 (20.2)	317 (31.2)		2.03 (1.74–2.36)	1.59 (1.34–1.88)	<0.001
Hardly ever/never		184 (2.7)	118 (2.1)	66 (6.7)	<0.001^a^	4.16 (3.04–5.69)	2.94 (2.05–4.21)	<0.001
Uses activities and resources independently	6,726							
Always/almost always		4,134 (61.5)	3,702 (64.5)	432 (43.7)		Ref		
Sometimes		2,254 (33.5)	1,797 (31.3)	457 (46.2)		2.18 (1.89–2.51)	1.74 (1.48–2.04)	<0.001
Hardly ever/never		338 (5.0)	238 (4.1)	100 (10.1)	<0.001^a^	3.59 (2.78–4.63)	2.52 (1.87–3.39)	<0.001
Interested, motivated, and excited to learn	6,724							
Always/almost always		4,379 (65.1)	3,918 (68.3)	461 (46.8)		Ref		
Sometimes		2,000 (29.7)	1,575 (27.4)	425 (43.1)		2.29 (1.98–2.65)	1.87 (1.59–2.21)	<0.001
Hardly ever/never		345 (5.1)	246 (4.3)	99 (10.1)	<0.001^a^	3.44 (2.67–4.42)	2.29 (1.70–3.10)	<0.001
Confident to try new things, initiate ideas and speak in familiar group	6,721							
Always/almost always		4,028 (59.9)	3,609 (62.9)	419 (42.5)		Ref		
Sometimes		2,193 (32.6)	1,785 (31.1)	408 (41.4)		1.97 (1.70–2.29)	1.71 (1.45–2.01)	<0.001
Hardly ever/never		500 (7.4)	342 (6.0)	158 (16.0)	<0.001^a^	3.99 (3.22–4.94)	2.73 (2.12–3.50)	<0.001
Maintains attention and concentrates	6,720							
Always/almost always		3,813 (56.7)	3,426 (59.8)	387 (39.2)		Ref		
Sometimes		2,420 (36.0)	1,950 (34.0)	470 (47.6)		2.13 (1.84–2.47)	1.75 (1.48–2.07)	<0.001
Hardly ever/never		487 (7.2)	356 (6.2)	131 (13.3)	<0.001^a^	3.25 (2.59–4.07)	2.42 (1.85–3.16)	<0.001
Perseveres when problem solving	6,714							
Always/almost always		3,676 (54.8)	3,324 (58.0)	352 (35.8)		Ref		
Sometimes		2,397 (35.7)	1,958 (34.2)	439 (44.7)		2.12 (1.82–2.46)	1.80 (1.52–2.14)	<0.001
Hardly ever/never		641 (9.5)	450 (7.9)	191 (19.5)	<0.001^a^	4.03 (3.29–4.93)	2.75 (2.17–3.50)	<0.001

a Pearson’s Chi-squared test.

b CI, Confidence Interval.

c Adjusted for child gender, gestational age, siblings, primary caregiver education, household income, parent–child relationship, child BMI, cultural background, child temperament, partner in household, maternal mental health.

### Teacher-reported emotional-behavioural function at age 9 years

As shown in Table 4, after adjustment for confounding variables children with BACA had significantly higher odds of having potentially problematic emotional-behavioural function (23.5% vs. 14.4%, AOR 1.34, 95% CI 1.10–1.63, *p* = 0.003). When the BACA group was split into those with a GCA score of 70–85 and those with GCA score < 70 (Supplementary Table S7), both groups remained significantly more likely to experience emotional-behavioural difficulties compared to their peers with TCD (GCA score 70–85 AOR 1.27, 95% CI 1.03–1.56; GCA score < 70 AOR 1.95, 95% CI 1.20–3.17). Neither group had higher odds of conduct difficulties compared to their peers with TCD. After adjustment for confounding, children with BACA had significantly higher odds of having teacher-reported difficulties with hyperactivity compared to their peers with TCD (24.6% vs. 14.9%, AOF 1.41, 95% CI 1.16–1.71).

**Table 4:** Teacher-reported emotional behavioural function at age 9 years.

	Valid	Total	Typical cognitive development	Below average cognitive ability	*p*-value	Odds ratio (95% CI^b^)	Adjusted odds ratio^c^ (95% CI)	*p*-value
Total Difficulties	6,738							
Average		5,680 (84.3)	4,923 (85.6)	757 (76.5)		Ref		
High		1,058 (15.7)	826 (14.4)	232 (23.5)	<0.001^a^	1.83 (1.55–2.15)	1.34 (1.10–1.63)	0.003
Emotional	6,739							
Average		5,631 (83.6)	4,853 (84.4)	778 (78.7)		Ref		
High		1,108 (16.4)	897 (15.6)	211 (21.3)	<0.001^a^	1.46 (1.24–1.73)	1.23 (1.02–1.49)	0.031
Conduct	6,739							
Average		6,118 (90.8)	5,242 (91.2)	876 (88.6)				
High		621 (9.2)	508 (8.8)	113 (11.4)	0.011^a^	1.34 (1.08–1.66)	0.92 (0.71–1.18)	0.507
Hyperactivity	6,738							
Average		5,641 (83.7)	4,895 (85.1)	746 (75.4)				
High		1,097 (16.3)	854 (14.9)	243 (24.6)	<0.001^a^	1.87 (1.59–2.20)	1.41 (1.16–1.71)	<0.001
Peer problems	6,739							
Average		5,799 (86.1)	5,000 (87.0)	799 (80.8)				
High		940 (13.9)	750 (13.0)	190 (19.2)	<0.001^a^	1.58 (1.33–1.89)	1.18 (0.96–1.45)	0.112
Prosocial^d^	6,737							
Average		5,888 (87.4)	5,062 (88.1)	826 (83.6)				
High		849 (12.6)	687 (11.9)	162 (16.4)	<0.001^a^	1.45 (1.20–1.75)	0.99 (0.79–1.23)	0.905

a Pearson’s Chi-squared test.

b CI, Confidence Interval.

c Adjusted for child gender, gestational age, siblings, primary caregiver education, household income, parent–child relationship, child BMI, cultural background, child temperament, partner in household, maternal mental health.

d The prosocial scale is a positive subscale but was reverse scored so that a higher score indicates potentially problematic function in this domain.

## Discussion

The aim of this study was to examine the causal association between below average cognitive ability at age 5, the age most children begin primary school in Ireland, and child-reported experience of school, child-reported self-concept, and teacher-reported class engagement and emotional-behavioural function at age 9. We found in a large nationally-representative sample, after adjustment for confounding, that children beginning primary school with BACA had higher odds of never liking school, of being picked on, of picking on others, and of reporting low self-concept. They had significantly higher odds of poor class engagement and problematic emotional-behavioural function, as reported by their teacher. The modelling approach, which involved careful consideration and stringent adjustment for confounding variables, provides strong evidence that early below average cognitive ability has a causal association with each adverse outcome. The socioeconomic environment to which the child was exposed, likely the most important confounding factor, was carefully adjusted for. In keeping with recommendations in the literature, collinearity between potential measures of SES was examined prior to inclusion of a range of measures representing different domains of the socioeconomic environment—parental education, family income, presence of a partner in the household, and the cultural background of the family (67).

There was strong evidence, after adjustment for confounding, of a causal relationship between BACA at age 5 and low overall self-concept at age 9. In particular, children with BACA had higher odds of low intellectual self-concept. Marsch’s widely published reciprocal-effects model proposes that low academic performance will adversely impact a child’s self-concept, which in turn will adversely affect a child’s future academic achievement, as the child is more likely to be hesitant to engage in learning, to fear failure, and to give up (69). The findings of this study, while limited to only two time points, lend support to this hypothesis. They confirm that children beginning school with BACA were at higher risk of low self-concept 4 years later, and were less likely to be excited to learn, to persevere when problem-solving, and to be confident to try new things, initiate ideas, or speak in the classroom. These findings have important implications for educational policy and practice and for the development of interventions. Marsh and Craven maintain that in order to enhance performance “enhancing skills alone is not enough; people also need to hold positive self-concepts of their abilities in specific areas.” (69)

Teachers were more likely to report poor classroom engagement for children with BACA, compared to their peers with typical cognitive development. Of note, they were no more likely to have conduct problems compared to their peers, suggesting disruptive behaviour did not contribute to their struggle to engage in the classroom. Children with BACA did however have difficulty with attention and concentration across a number of measures. They were significantly more likely to hardly ever or never maintain attention and concentration in the classroom compared to peers with TCD (13.3% vs. 6.2%), and were significantly more likely to have teacher-reported hyperactivity difficulties (24.6% vs. 14.9%). While one could argue that these children may have other underlying diagnoses, such as attention deficit hyperactivity disorder (ADHD) or autism spectrum disorder (ASD), at age 9 years only 7% of all children with BACA had teacher-reported limitations in learning due to a diagnosis of an emotional-behavioural disorder such as ADHD, and only 5% due to a diagnosis of ASD. This does not rule out undiagnosed disorders, including specific learning disabilities, such as dyslexia or dyscalculia, which may or may not be accompanied by lower cognitive ability. However, for some children with BACA the attention and concentration difficulties could reflect an inability to engage and keep up on a cognitive level with the activities taking place in the classroom.

It is unsurprising that children with BACA had higher odds of never liking school, a sentiment which tends to persist over time, and which increases the risk of absenteeism and early school dropout (70, 71). Early school dropout itself is a risk factor for incarceration, substance use, teenage pregnancy and poor health (72).

While cognitive ability alone does not determine a child’s success, happiness, or achievement (73), the results of this study clearly show it has an association with a child’s ability to engage in learning, with their appraisal of themselves, and with other aspects of their emotional and behavioural functioning. There is a risk of placing undue emphasis on a child’s cognitive ability, and failing to recognise and foster other forms of ability and intelligence, such as musical, interpersonal, and bodily-kinaesthetic intelligence, however it cannot be denied that a child’s cognitive ability is an important determinant of outcomes throughout the lifecourse (74). If every child is to benefit equally from their right to education, a multi-faceted, inter-agency approach which looks at cognition from a primary, secondary, and tertiary prevention approach is required.

Every child should have the opportunity to reach their cognitive potential. For some children, genetic conditions at birth may result in a lower potential cognitive ability, but for many children it is the environment into which they are born which prevents them reaching their cognitive potential. In this study, a significantly higher proportion of children with BACA were from lower socioeconomic backgrounds, a finding consistent across the literature (75). Infants born into socioeconomic adversity are more likely to experience parental depression, homelessness, and drug or alcohol abuse. They may not receive adequate interaction and support, the basic prerequisites for cognitive development. They may not have access to the tangible resources, for example books and toys, and intangible resources, for example family social capital, that promote optimal cognitive development (76). Political commitment, investment, and social policy reform are required to address homelessness, deprivation, unemployment, and addiction. Without this, achieving educational equality will remain an uphill struggle.

Early intensive interventions, which begin in infancy, focus on families as well as the infant, and provide developmentally enriching environments, can improve cognitive outcomes for at risk children (77). The challenge lies in identifying, in a timely fashion, who these children are. Some countries, such as the United States, adopt a risk-based approach and provide population-based early intervention on the basis of socioeconomic risk factors (77). Other countries have implemented standardised assessment at school-entry, however this has the potential to systematically delay school-starting age for children from disadvantaged backgrounds and risks further stigmatisation (78). Additionally, this approach would only allow interventions to be started at the time of expected school entry, when significant differences in cognitive ability already exist. The ideal time for intervention is in very early life, when the brain is at its optimal stage of neuroplasticity. Current routinely used developmental screening tools, such as the Ages and Stages Questionnaire, will not detect the majority of children with BACA at school-age and cannot therefore be used to achieve this (79, 80). There is a need for alternative methods of early identification for children at risk of poor cognitive outcomes at school-age. In Ireland, there is currently no population-based programme of early intervention for children at risk of poor cognitive outcomes. As alluded to previously, designing such an intervention should acknowledge the reciprocal relationship between cognitive ability and self-concept, and should aim to enhance both simultaneously.

Finally, from a tertiary prevention perspective, all children will begin their educational journey with different strengths and weaknesses, with different skills and abilities, and with different aspirations for what they wish to gain from their education. Every child should have equal opportunity to fulfil these aspirations in a supportive, bias-free system, which values both cognitive and non-cognitive skills. In Ireland, and in many countries internationally, this may mean shifting the focus from solely one of cognitive achievement, and adapting both teaching and assessment methods to incorporate other forms of ability and achievement (81).

Major strengths of this study include the large sample size, the careful sampling strategy employed in recruitment of the cohort, and the application of a weighting variable to all analyses to account for non-response and attrition, all of which enable us to make relatively confident inferences about the population from which the sample was drawn.

## Limitations

Cognitive ability was directly measured using standardised assessments. Children taking the naming vocabulary test, for whom English is not their native language, may be disadvantaged, with apparent poor performance. The British Ability Scales is a valid tool for measuring general cognitive ability, and is comparable to measures of IQ such as the Wechsler Intelligence Test (82). Children in GUI did not complete all BAS subtests and PCA was therefore used to calculate a GCA score. This approach would not be acceptable for a formal diagnosis of an IDD.

Efforts were made in GUI to minimise response bias through the use of anonymous self-complete questionnaires for sensitive topics. While the application of a weighting variable aimed to minimise the overall effect of non-response and attrition on the structure of the cohort, selective missing data remains a source of bias. For example, teacher-reported missing data for individual students may be systematically more likely in schools which are under-resourced. Missing data were treated with complete case analyses, a further source of bias.

Teacher-reported outcomes were used for assessing the child’s classroom engagement and their emotional-behavioural function. It is well-documented that teacher-reported outcomes are subject to a teacher’s implicit bias. A teacher’s assessment of a child may be affected by the child’s race, socioeconomic background, gender, or other characteristic (83). Their treatment of the child can result in a self-fulfilling prophecy whereby the teacher’s expectations of the student influence the actual performance and academic achievement of the student (84).

The study did not adjust for the presence of underlying conditions at age 5 which may affect cognitive ability, such as ASD and ADHD, as this data was not available. However, data from the at 9 year survey would suggest that a minority of children in the BACA group were diagnosed with these conditions.

## Conclusion

Acknowledging these limitations, this study shows that for children with BACA at the beginning of school, the odds of experiencing low child-reported self-concept, poor teacher-reported classroom engagement, and problematic teacher-reported emotional-behavioural function are significantly higher. The findings support the hypothesis that cognitive ability itself has a causal relationship with these outcomes.

For families, the findings of this study suggest that if a child is disengaging from school; has a negative self-perception, particularly of their intellectual ability; is displaying, or is reported by their teacher to be displaying, problematic emotions or behaviours, then consideration should be given to the child’s cognitive functioning as a potential contributing factor. Families and teachers, should together determine in these scenarios whether further exploration of the child’s cognitive functioning is warranted. Teachers should have access, without undue delay or administrative barriers, to psychological assessments and to supports such as mental health services, speech and language therapists, psychological services, and learning support if they are necessary.

Every child should have the opportunity to reach their individual cognitive potential. Reducing socioeconomic inequality is crucial in this regard. Early intervention programmes can improve cognitive ability but further research is required on the optimal method to identify which children will benefit most. Finally, adapting our education system to provide support and resources for those with lower cognitive potential, and to foster and promote other types of non-cognitive abilities is essential.

## Data Availability

The data analysed in this study is subject to the following licenses/restrictions: researchers may apply for access to the dataset by submitting an application to ISSDA. Requests to access these datasets should be directed to www.ucd.ie/issda.
