# Supplementary material for: The relationship between below average cognitive ability at age 5 years and the child’s experience of school at age 9
Source: Front Public Health. 2025 Mar 4;13:1341797. doi: 10.3389/fpubh.2025.1341797 (PMC11913694; doi:10.3389/fpubh.2025.1341797)
Supplement: Supplementary file 1 [file Table_1.docx]

***Supplementary Material***

**Title: The impact of below average cognitive ability at age 5 years on childhood experience of school at age 9**

**Andrea K Bowe^1*^, Mathias Urban^2^, Anthony Staines^3^, Deirdre M Murray^1,4^**

**^*^ Correspondence: Andrea Bowe: abowe@ucc.ie**

Supplemental Table S1. Child and teacher questions and potential responses

| **Respondent** | **Format of questionnaire** | **Age** | **Question** | **Potential Responses** | **Scoring or recoding if relevant** |
| --- | --- | --- | --- | --- | --- |
| **Covariates** |  |  |  |  |  |
| Parent | Self-complete^a^ | 9 months | **Center for Epidemiological Studies Depression Scale (CES-D)**   1. I felt I could not shake off the blues even with help from my family or friends 2. I felt depressed 3. I thought my life had been a failure 4. I felt fearful 5. My sleep was restless 6. I felt lonely 7. I had crying spells 8. I felt sad | Rarely or none of the time (less than 1 day) (0)  Some or a little of the time (1-2 days) (1)  Occasionally or a moderate amount of the time (3-4 days) (2)  Most or all of the time (5-7 days) (3) | Score range 0-24 |
| Parent | Interviewer administered^b^ | 9 months | After how many weeks of pregnancy was baby born? | 25 – 25 weeks or less Otherwise numeric |  |
| Parent | Interviewer administered | 9 months | Can you tell me what is your ethnic or cultural background? | Irish  Other white  African/other black  Chinese/other Asian  Other |  |
| Parent | Self-complete | 5 years | **Adapted Short Temperament Scale**   1. This child is shy with strange adults 2. When this child starts a project such as a puzzle or model, he/she works on it without stopping until it is completed, even if it takes a long time 3. If this child wants a toy or sweet while shopping, he/she will easily accept something else instead 4. This child is shy when first meeting new children 5. This child likes to complete one task or activity before going onto the next 6. When this child is angry about something, it is difficult to side-track him/her 7. When in a park or visiting, this child will go up to strange children and join in their play 8. This child stays with an activity(e.g. puzzle, construction kit, reading) for a long time 9. When shopping together, if I do not buy what this child wants (e.g. sweets, clothing) he/she cries and yells 10. When unknown adults visit our home, this child is immediately friendly and approaches them 11. If this child is upset, it is hard to comfort him/her 12. When a toy or game becomes difficult, this child quickly turns to another activity | Almost never (1)  Not often (2)  Variable, usually does not (3)  Variable usually does (4)  Frequently (5)  Almost always (6) | Each of the three subscales of sociability, persistence and reactivity consists of 4-items scored from 1-6. The average score from the four items is used as the score for that subscale. |
| Parent | Interviewer administered | 5 years | Do you have a spouse/partner who lives here with you in household? | Yes  No |  |
| Parent | Interviewer administered | 5 years | Does child have any brothers or sisters? | Yes  No |  |
| Parent | Interviewer administered | 5 years | What is the highest level of education (full-time or part-time) which you have completed to date? | 1. No formal education 2. Primary education 3. Lower secondary 4. Upper secondary 5. Technical or vocational qualification 6. National certificate, Diploma, Nursing Diploma (Non-Degree) 7. Primary Degree 8. Professional Qualification of at least degree status 9. Both a degree and a professional qualification 10. Postgraduate certificate or diploma 11. Postgraduate degree (Masters) 12. Postgraduate doctorate (PhD) | Lower secondary or less (a,b,c)  Secondary complete (d)  Technical or vocational (e)  Certificate or diploma (f)  Primary degree (g,h,i)  Post degree qualification (j,k,l) |
| Parent | Derived | 5 years | **Household social class**  A social class classification was generated for both the primary and secondary caregiver based on details they provided in the questionnaire about their occupation from current or previous employment. The household’s social class was taken as the highest social class category of both caregivers in the household. Where both caregivers were economically inactive and had not had any previous employment they were classified as ‘validly no social class’. | 1. Professional workers  2. Managerial and technical  3. Non-manual  4. Skilled manual  5. Semi-skilled  6. Unskilled  7. All others gainfully occupied and unknown  8. Validly no social class |  |
| Parent | Derived | 5 years | **Equivalised household income**  An equivalence scale was used to assign a weight of 1 to the first adult in the household, and a weight of 0.66 to each subsequent adult (age 14+) living in the household, with a weight of 0.33 assigned to each child (age less than 14). The sum of the weights was used as the equivalised size. Disposable household income, recorded as total gross household income reported less statutory deductions of income tax and social insurance contributions, was divided by the equivalised size to provide a measure of equivalised household income. |  | Quintiles  Range 1-5 (Lowest – Highest) |
| Measured | Derived | 5 years | **Child BMI**  The child’s weight was measured by the interviewer using a medically approved digital scale and height was measured using a standardised measuring stick. BMI was derived from these variables. | Numeric |  |
| Parent | Self-complete | 5 years | **Pianta Child Parent Relationship Scale**   1. I share an affectionate, warm relationship with my child 2. My child and I always seem to be struggling with each other 3. If upset, my child will seek comfort from me 4. My child is uncomfortable with physical affection or touch from me 5. My child values his/her relationship with me 6. When I praise my child he/she beams with pride 7. My child spontaneously shares information about his/herself 8. My child easily becomes angry at me 9. It is easy to be in tune with what my child is feeling 10. My child remains angry or is resistant after being disciplined 11. Dealing with my child drains my energy 12. When my child is in a bad mood I know we’re in for a long and difficult day 13. My child’s feelings toward me can be unpredictable or can change suddenly 14. My child is sneaky or manipulative with me 15. My child openly shares his/her feelings and experiences with me | Definitely does not apply (1)  Does not really apply (2)  Neutral not sure (3)  Applies somewhat (4)  Definitely applies (5) | Positive subscale score range 8-40  Conflict subscale score range 8-40 |
| Teacher | Posted questionnaire | 5 years | Do any of the following limit the kind or amount of activity the Study Child can do at school?   1. Physical disability or visual or hearing impairment 2. Speech impairment 3. Autism spectrum disorder 4. General learning disability: mild 5. General learning disability: moderate/severe/profound 6. Specific learning difficulties (e.g. dyslexia) 7. Emotional or behavioural problem (e.g. Attention Deficit Hyperactivity Disorder) 8. Home environment/problems at home 9. Has limited knowledge of the main language of instruction 10. Discipline problems 11. Poor attendance 12. Other | Yes  No |  |
| Teacher | Posted questionnaire | 5 years | If answered ‘yes’ to any of the above – Does the study child receive special help or resources in the school because of this (these) limitations? | Yes  No |  |
| Teacher | Posted questionnaire | 9 years | Do any of the following limit the kind or amount of activity the Study Child can do at school?   1. Physical disability or visual or hearing impairment 2. Speech impairment 3. Autism spectrum disorder 4. General learning disability: mild 5. General learning disability: moderate/severe/profound 6. Specific learning difficulties (e.g. dyslexia) 7. Emotional or behavioural problem (e.g. Attention Deficit Hyperactivity Disorder) 8. Home environment/problems at home 9. Has limited knowledge of the main language of instruction 10. Discipline problems 11. Poor attendance 12. Other | Yes  No |  |
| **Outcome variables** |  |  |  |  |  |
| Child | Interviewer administered^c^ | 9 years | What do you think about school? | Always like it  Sometimes like it  Never like it |  |
| Child | Interviewer administered | 9 years | How well do you think you are doing in your school work? | Well  Average/Ok  Poorly |  |
| Child | Self-complete^d^ | 9 years | Thinking back over the last year would you say that anyone (either a child or an adult) picked on you? | Yes  No |  |
| Child | Self-complete | 9 years | Thinking back over the last year would you say that you picked on someone (either a child or an adult) | Yes  No |  |
| Child | Self- complete | 9 years | Piers-Harris Self-Concept Scale  Items cannot be published due to copyright | Yes  No | ‘Very low’, ‘low, ‘low average’ categorised as ‘Low’.  ‘Average’, ‘high average’, ‘high’ categorised as ‘Average or high’. |
| Teacher | Posted questionnaire | 9 years | In general would you say the study child shows an interest in classroom activities through observation or participation | Always/almost always  Sometimes  Hardly ever/Never |  |
| Teacher | Posted questionnaire | 9 years | In general would you say the study child displays high levels of involvement in self-chosen activities? | Always/almost always  Sometimes  Hardly ever/Never |  |
| Teacher | Posted questionnaire | 9 years | In general would you say the study child selects and uses activities and resources independently? | Always/almost always  Sometimes  Hardly ever/Never |  |
| Teacher | Posted questionnaire | 9 years | In general would you say the study child continues to be interested, motivated, and excited to learn? | Always/almost always  Sometimes  Hardly ever/Never |  |
| Teacher | Posted questionnaire | 9 years | In general would you say the study child is confident to try new activities, initiate ideas, and to speak in a familiar group? | Always/almost always  Sometimes  Hardly ever/Never |  |
| Teacher | Posted questionnaire | 9 years | In general would you say the study child maintains attention and concentrates? | Always/almost always  Sometimes  Hardly ever/Never |  |
| Teacher | Posted questionnaire | 9 years | In general would you say the study child sustains involvement and perseveres, particularly when trying to solve a problem or reach a satisfactory conclusion? | Always/almost always  Sometimes  Hardly ever/Never |  |
| Teacher | Posted questionnaire | 9 years | Strengths and Difficulties Questionnaire Items. Listed below is a set of statements which could be used to describe the Study Child’s behaviour. For each item please mark box….   1. Considerate of other people’s feelings 2. Restless, overactive, cannot stay still for long 3. Often complains of headaches, stomach-aches or sickness 4. Shares readily with other children (treats, toys, pencils, etc) 5. Often has temper tantrums or hot tempers 6. Rather solitary, tends to play alone 7. Generally obedient, usually does what adults request 8. Many worries, often seems worried 9. Helpful if someone is hurt, upset or feeling ill 10. Constantly fidgeting or squirming 11. Has at least one good friend 12. Often fights with other children or bullies them 13. Often unhappy, downhearted or tearful 14. Generally liked by other children 15. Easily distracted, concentration wanders 16. Nervous or clingy in new situations, easily loses confidence 17. Kind to younger children 18. Often lies or cheats 19. Picked on or bullied by other children 20. Often volunteers to help others (parents, teachers, other children) 21. Thinks things out before acting 22. Steals from home, school or elsewhere 23. Gets on better with adults than with other children 24. Many fears, easily scared 25. Sees tasks through to the end, good attention span | Not true  Somewhat True  Certainly True | Total difficulties ≥12 – High  Emotional ≥4 – High  Conduct ≥3 – High  Hyperactivity ≥6 – High  Peer problems ≥3 – High  Prosocial ≤5 - High |

^a^ Computer assisted self-completion interview was used for self-complete parental questionnaires, ^b^ Computer assisted personal interviewing was used for interviewer administered parent questionnaires, ^c^ Computer assisted personal interviewing was used for interviewer administered child questionnaires, ^d^ Self-complete child questionnaires were completed using a paper-based questionnaire

Supplemental Table S2. Characteristics of study population and comparison of characteristics between those with typical cognitive development, below average cognitive ability, and low cognitive ability.

|  | Valid | Total  n=7,392 | Typical cognitive development  (GCA ≥ 85)  n=6,286 | Below average cognitive ability  (GCA 70-85)  n=952 | Low cognitive ability  (GCA <70)  n=154 | p-value |
| --- | --- | --- | --- | --- | --- | --- |
| **Child characteristics** |  |  |  |  |  |  |
| Gender | 7,392 |  |  |  |  |  |
| Male |  | 3,783 (51.2) | 3,131 (49.8) | 562 (59.1) | 89 (57.8) | <0.001^a^ |
| Gestational age | 7,375 |  |  |  |  |  |
| N, mean (sd^b^) |  | 39.5 (2.1) | 6,277, 39.5 (2.1) | 949, 39.6 (2.1) | 149, 39.4 (2.6) | 0.609^c^ |
| Child temperament |  |  |  |  |  |  |
| Sociability – n, median (IQR^d^) | 7,389 | 4.0 (3.0-5.0) | 6,285, 4.0 (3.0-5.0) | 950, 3.8 (3.0-4.8) | 153, 4.0 (2.5-5.0) | 0.050^d^ |
| Persistence – n, median (IQR^d^) | 7,373 | 4.3 (3.5-5.0) | 6,279, 4.3 (3.5-5.0) | 947, 4.0 (3.0-4.8) | 147, 3.8 (2.8-4.8) | <0.001^d^ |
| Reactivity – n, median (IQR^d^) | 7,381 | 2.3 (1.8-3.0) | 6,282, 2.3 (1.8-3.0) | 950, 2.3 (1.8-3.0) | 148, 2.8 (2.0-3.8) | <0.001^d^ |
| General cognitive ability score | 7,392 |  |  |  |  |  |
| N, mean (sd) |  | 99.8 (14.9) | 6,286, 103.8 (12.1) | 951, 79.2 (4.2) | 154, 64.6 (5.5) | <0.001^c^ |
| Body mass index – kg/m^2^ | 7,319 | 16.3 (1.7) | 6,231, 16.3 (1.7) | 936, 16.3 (1.7) | 152, 16.8 (2.1) | 0.002^c^ |
| **Sociodemographic characteristics** |  |  |  |  |  |  |
| Partner in household | 7,392 |  |  |  |  |  |
| Yes |  | 6,224 (84.2) | 5,352 (85.1) | 746 (78.4) | 126 (81.8) | <0.001^a^ |
| Siblings | 7,391 |  |  |  |  |  |
| Yes |  | 6,479 (87.7) | 5,514 (87.7) | 836 (87.9) | 129 (83.8) | 0.328^a^ |
| Cultural background | 7,375 |  |  |  |  |  |
| Irish |  | 6,398 (86.8) | 5,598 (89.1) | 717 (75.9) | 83 (56.5) |  |
| Other white |  | 642 (8.7) | 457 (7.3) | 149 (15.8) | 37 (25.2) |  |
| African or other black |  | 156 (2.1) | 93 (1.5) | 55 (5.8) | 8 (5.4) |  |
| Chinese or other Asian |  | 151 (2.0) | 116 (1.8) | 21 (2.2) | 15 (10.2) |  |
| Other |  | 28 (0.4) | 20 (0.3) | 3 (0.3) | 4 (2.7) | <0.001^a^ |
| PCG Highest education | 7,392 |  |  |  |  |  |
| Lower secondary or less |  | 965 (13.1) | 722 (11.5) | 214 (22.5) | 29 (18.8) |  |
| Secondary |  | 1,227 (16.6) | 1,027 (16.3) | 169 (17.8) | 31 (20.1) |  |
| Technical or vocational |  | 1,430 (19.3) | 1,169 (18.6) | 217 (22.8) | 45 (29.2) |  |
| Certificate or diploma |  | 1,605 (21.7) | 1,421 (22.6) | 163 (17.1) | 21 (13.6) |  |
| Primary degree |  | 851 (11.5) | 746 (11.9) | 88 (9.2) | 17 (11.0) |  |
| Post degree qualification |  | 1,313 (17.8) | 1,201 (19.1) | 101 (10.6) | 11 (7.1) | <0.001^a^ |
| Household social class | 7,392 |  |  |  |  |  |
| Professional workers |  | 934 (12.6) | 858 (13.6) | 63 (6.6) | 13 (8.4) |  |
| Managerial and technical |  | 2,380 (32.2) | 2,164 (34.4) | 191 (20.1) | 24 (15.6) |  |
| Non-manual |  | 1,334 (18.0) | 1,126 (17.9) | 186 (19.5) | 22 (14.3) |  |
| Skilled manual |  | 1,115 (15.1) | 868 (13.8) | 213 (22.4) | 34 (22.1) |  |
| Semi-skilled |  | 668 (9.0) | 525 (8.4) | 126 (13.2) | 18 (11.7) |  |
| Unskilled |  | 110 (1.5) | 95 (1.5) | 15 (1.6) | 0 (0.0) |  |
| All others gainfully occupied |  | 76 (1.0) | 51 (0.8) | 19 (2.0) | 6 (3.9) |  |
| Validly no social class^b^ |  | 775 (10.5) | 599 (9.5) | 139 (14.6) | 37 (24.0) | <0.001^a^ |
| Equivalised Income Quintile^c^ | 7,009 |  |  |  |  |  |
| Lowest |  | 1,448 (20.7) | 1,107 (18.5) | 286 (32.1) | 55 (39.9) |  |
| 2 |  | 1,472 (21.0) | 1,194 (20.0) | 238 (26.7) | 40 (29.0) |  |
| 3 |  | 1,383 (19.7) | 1,207 (20.2) | 150 (16.8) | 26 (18.8) |  |
| 4 |  | 1,360 (19.4) | 1,231 (20.6) | 118 (13.2) | 11 (8.0) |  |
| Highest |  | 1,346 (19.2) | 1,240 (20.7) | 100 (11.2) | 6 (4.3) | <0.001^a^ |
| **Relationship characteristics** |  |  |  |  |  |  |
| Parent-child relationship |  |  |  |  |  |  |
| Closeness – n, median (IQR) | 7,380 | 35.0 (33.0-35.0) | 6,285, 35.0 (33.0-35.0) | 947, 34.0 (33.0-35.0) | 148, 34.0 (32.0-35.0) | <0.001^e^ |
| Conflict – n, median (IQR) | 7,384 | 14.0 (10.0-19.0) | 6,284, 14.0 (10.0-18.0) | 951, 15.0 (11.0-19.0) | 149, 16.0 (11.0-21.0) | <0.001^e^ |

^a^ Pearson’s Chi-squared test, ^b^ Standard deviation, ^c^ Analysis of variance (ANOVA), ^d^ Interquartile range, ^e^ Independent samples Kruskal-Wallis test,

Validly no social class consists of households where both caregivers are currently economically inactive and have not held any previous employment in the past, ^c^ Equivalised Income Quintile is based on the disposable household income (total gross income less statutory deductions), divided by the equivalised household size which takes account of differences in size and composition of households ^d^ Independent samples t-test, ^e^ sd – standard deviation.

Supplemental Table S3. Learning limitations and supports reported by child’s teacher at age 5 and age 9

|  | Valid | Total  n=7,392 | Typical cognitive development  (GCA ≥ 85)  n=6,286 | Below average cognitive ability  (GCA 70-85)  n=952 | Low cognitive ability  (GCA <70)  n=154 | p-value |
| --- | --- | --- | --- | --- | --- | --- |
| Recognised limitation to learning at age 5 | 6,955 |  |  |  |  |  |
| No |  | 5,420 (77.9) | 4,854 (81.9) | 525 (59.1) | 41 (29.5) |  |
| Yes |  | 1,535 (22.1) | 1,074 (18.1) | 363 (40.9) | 98 (70.5) | <0.001^a^ |
| Special help or resources provided to child at age 5 | 6,954 |  |  |  |  |  |
| No |  | 6,132 (88.2) | 5,394 (91.0) | 676 (76.1) | 62 (44.6) |  |
| Yes |  | 822 (11.8) | 533 (9.0) | 212 (23.9) | 77 (55.4) | <0.001^a^ |
| Specific limitations to learning at age 9 |  |  |  |  |  |  |
| Physical/visual/hearing impairment | 6,491 | 177 (2.7) | 136 (2.5) | 32 (3.9) | 9 (7.0) | <0.001^a^ |
| Speech impairment | 6,487 | 155 (2.4) | 103 (1.9) | 37 (4.5) | 15 (11.7) | <0.001^a^ |
| Autism spectrum disorder | 6,468 | 205 (3.2) | 149 (2.7) | 38 (4.6) | 18 (14.2) | <0.001^a^ |
| Specific learning difficulty e.g. dyslexia | 6,439 | 428 (6.6) | 331 (6.0) | 76 (9.3) | 21 (16.5) | <0.001^a^ |
| General learning disability - mild | 6,457 | 324 (5.0) | 213 (3.9) | 89 (10.9) | 22 (17.5) | <0.001^a^ |
| General learning disability – mod/sev/profound | 6,444 | 80 (1.2) | 53 (1.0) | 17 (2.1) | 10 (7.8) | <0.001^a^ |
| Emotional or behavioural problem e.g. ADHD^b^ | 6,443 | 261 (4.1) | 198 (3.6) | 50 (6.1) | 12 (10.2) | <0.001^a^ |
| Home environment | 6,137 | 326 (5.0) | 239 (4.3) | 79 (9.6) | 8 (6.3) | <0.001^a^ |
| Language | 6,476 | 128 (2.0) | 51 (0.9) | 53 (6.4) | 24 (18.9) | <0.001^a^ |
| Discipline | 6,468 | 192 (3.0) | 144 (2.6) | 43 (5.2) | 5 (3.9) | <0.001^a^ |
| Poor attendance | 6,464 | 214 (3.3) | 145 (2.6) | 60 (7.3) | 9 (7.0) | <0.001^a^ |
| Other | 5,633 | 297 (5.3) | 253 (5.2) | 38 (5.5) | 6 (6.3) | 0.872^a^ |

^a^ Pearson’s Chi-squared test


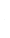
Supplemental Table S4. Child-reported experience of school at age 9

|  | Valid | Total | Typical cognitive development  (GCA ≥ 85)  n=6,286 | Below average cognitive ability  (GCA 70-85)  n=952 | Low cognitive ability  (GCA <70)  n=154 | p-value | BACA^a^ v TCD^b^  Odds ratio  (95% CI^e^) | BACA v TCD  Adjusted odds ratio^f^ (95% CI^e^) | LCA^c^ v TCD  Odds ratio  (95% CI^e^) | LCA v TCD  Adjusted odds ratio^f^ (95% CI^e^) |
| --- | --- | --- | --- | --- | --- | --- | --- | --- | --- | --- |
| **Thoughts about school** | 7,305 |  |  |  |  |  |  |  |  |  |
| Always like it |  | 2,409 (33.3) | 2,088  (33.6) | 275  (29.1) | 46  (31.5) |  | Ref | Ref | Ref | Ref |
| Sometimes like it |  | 4,494 (61.5) | 3,837  (61.7) | 581  (61.5) | 76  (52.1) |  | 1.15  (0.99-1.34) | 1.06  (0.89-1.25) | 0.90  (0.62-1.31) | 0.67  (0.43-1.05) |
| Never like |  | 402 (5.5) | 290  (4.7) | 88  (9.3) | 24  (16.4) | <0.001^d^ | 2.29  (1.75-3.00) | 1.74  (1.28-2.37) | 3.71  (2.22-6.19) | 2.30  (1.20-4.38) |
| **School performance** | 7,298 |  |  |  |  |  |  |  |  |  |
| Well |  | 4,013 (55.0) | 3,488  (56.2) | 448  (47.5) | 77  (53.5) |  | Ref | Ref | Ref | Ref |
| Average |  | 3,195 (43.8) | 2,668  (43.0) | 468  (49.6) | 59  (41.0) |  | 1.36  (1.19-1.57) | 1.41  (1.21-1.65) | 1.00  (0.71-1.41) | 1.05  (0.70-1.58) |
| Poorly |  | 90 (1.2) | 54  (0.9) | 28  (3.0) | 8  (5.6) | <0.001^a^ | 3.95  (2.47-6.31) | 2.96  (1.77-4.96) | 6.93  (3.23-14.84) | 3.81  (1.21-12.00) |
| **Picked on in past year** | 6,978 |  |  |  |  |  |  |  |  |  |
| No |  | 4,111 (58.8) | 3,573  (59.9) | 466  (53.1) | 71  (53.8) |  | Ref | Ref | Ref | Ref |
| Yes |  | 2,867 (41.1) | 2,394  (40.1) | 412  (46.9) | 61  (46.2) | <0.001^a^ | 1.32  (1.14-1.52) | 1.31  (1.12-1.53) | 1.27  (0.90-1.79) | 0.99  (0.64-1.53) |
| **Picked on other in past year** | 6,872 |  |  |  |  |  |  |  |  |  |
| No |  | 5,819 (84.7) | 5,045  (86.0) | 681  (78.0) | 92  (70.8) |  | Ref | Ref | Ref | Ref |
| Yes |  | 1,053 (15.3) | 822  (14.0) | 192  (22.0) | 38  (29.2) | <0.001^a^ | 1.73  (1.45-2.07) | 1.48  (1.22-1.80) | 2.55  (1.74-3.75) | 2.04  (1.24-3.35) |

^a^ Below average cognitive ability, ^b^ Typical cognitive development, ^c^ Low cognitive ability, ^d^ Pearson’s Chi-squared test, ^e^ CI - Confidence Interval, ^f^ Adjusted for child gender, gestational age, siblings, primary caregiver education, household income, parent-child relationship, child BMI, cultural background, child temperament, partner in household, maternal mental health

Supplemental Table S5. Child’s self-concept at age 9 years

|  | Valid | Total | Typical cognitive development  (GCA ≥ 85)  n=6,286 | Below average cognitive ability  (GCA 70-85)  n=952 | Low cognitive ability  (GCA <70)  n=154 | p-value | BACA^a^ v TCD^b^  Odds ratio  (95% CI^e^) | BACA v TCD  Adjusted odds ratio^f^ (95% CI^e^) | LCA ^c^v TCD  Odds ratio  (95% CI^e^) | LCA v TCD  Adjusted odds ratio^f^ (95% CI^e^) |
| --- | --- | --- | --- | --- | --- | --- | --- | --- | --- | --- |
| **Overall self-concept** | 6,702 |  |  |  |  |  |  |  |  |  |
| Average or high |  | 4,860 (72.5) | 4,236  (73.8) | 568  (67.5) | 56  (45.2) |  | Ref | Ref | Ref | Ref |
| Low |  | 1,842 (27.5) | 1,501  (26.2) | 273  (32.5) | 68  (54.8) | <0.001^d^ | 1.36  (1.16-1.59) | 1.12  (0.94-1.34) | 3.39  (2.37-4.85) | 2.17  (1.38-3.41) |
| **Intellectual and school status** | 6,671 |  |  |  |  |  |  |  |  |  |
| Average or high |  | 4,960 (74.4) | 4,335  (75.9) | 563  (67.5) | 62  (50.0) |  | Ref | Ref | Ref | Ref |
| Low |  | 1,711 (25.6) | 1,378  (24.1) | 271  (32.5) | 62  (50.0) | <0.001^d^ | 1.51  (1.29-1.77) | 1.30  (1.09-1.55) | 3.15  (2.21-4.50) | 2.25  (1.44-3.52) |
| **Behavioural adjustment** | 6,845 |  |  |  |  |  |  |  |  |  |
| Average^g^ |  | 4,388 (64.1) | 3,841  (65.6) | 496  (57.9) | 51  (39.2) |  | Ref | Ref | Ref | Ref |
| Low |  | 2,457 (35.9) | 2,018  (34.4) | 360  (42.0) | 79  (60.8) | <0.001^d^ | 1.38  (1.19-1.60) | 1.18  (1.00-1.38) | 2.96  (2.08-4.23) | 2.42  (1.55-3.79) |
| **Physical appearance and attributes** | 6,799 |  |  |  |  |  |  |  |  |  |
| Average |  | 5,155 (75.8) | 4,449  (76.6) | 628  (72.8) | 78  (61.4) |  | Ref | Ref | Ref | Ref |
| Low |  | 1,644 (24.2) | 1,360  (23.4) | 235  (27.2) | 49  (38.6) | <0.001^d^ | 1.23  (1.04-1.44) | 1.12  (0.94-1.34) | 2.06  (1.43-2.96) | 1.64  (1.04-2.60) |
| **Freedom from anxiety** | 6,878 |  |  |  |  |  |  |  |  |  |
| Average or high |  | 5,317 (77.3) | 4,584  (77.9) | 654  (75.4) | 79  (60.8) |  | Ref | Ref | Ref | Ref |
| Low |  | 1,561 (22.7) | 1,297  (22.1) | 213  (24.6) | 51  (39.2) | <0.001^d^ | 1.15  (0.97-1.36) | 0.99  (0.82-1.19) | 2.30  (1.61-3.28) | 1.40  (0.88-2.23) |
| **Popularity** | 6,810 |  |  |  |  |  |  |  |  |  |
| Average or high |  | 5,214 (76.6) | 4,524  (77.7) | 620  (72.2) | 70  (54.3) |  | Ref | Ref | Ref | Ref |
| Low |  | 1,596 (23.4) | 1,298  (22.3) | 239  (27.8) | 59  (45.7) | <0.001^d^ | 1.34  (1.14-1.58) | 1.12  (0.94-1.34) | 2.96  (2.08-4.20) | 2.20  (1.41-3.42) |
| **Happiness and satisfaction** | 6,877 |  |  |  |  |  |  |  |  |  |
| Average^g^ |  | 4,562 (66.3) | 3,997  (68.0) | 518  (59.9) | 47  (35.9) |  | Ref | Ref | Ref | Ref |
| Low |  | 2,315 (33.7) | 1,885  (32.0) | 347  (40.1) | 84  (64.1) | <0.001^d^ | 1.42  (1.23-1.64) | 1.17  (1.00-1.38) | 3.80  (2.65-5.46) | 2.96  (1.90-4.62) |

^a^ Below average cognitive ability, ^b^ Typical cognitive development, ^c^ Low cognitive ability, ^d^ Pearson’s Chi-squared test, ^e^ CI - Confidence Interval, ^f^ Adjusted for child gender, gestational age, siblings, primary caregiver education, household income, parent-child relationship, child BMI, cultural background, child temperament, partner in household, maternal mental health, ^g^ Where ‘Average’ is listed alone no child score in the ‘high average’ or ‘high’ categories

Supplemental Table S6. Teacher-reported classroom engagement and behaviour at age 9 years

|  | Valid | Total | TCD^a^  (GCA ≥ 85)  n=6,286 | BACA^b^  (GCA 70-85)  n=952 | LCA^c^  (GCA <70)  n=154 | p-value | BACA v TCD  Odds ratio  (95% CI^e^) | BACA v TCD  Adjusted odds ratio^f^ (95% CI^e^) | LCA v TCD  Odds ratio  (95% CI^e^) | LCA v TCD  Adjusted odds ratio^f^ (95% CI^e^) |
| --- | --- | --- | --- | --- | --- | --- | --- | --- | --- | --- |
| **Shows an interest in classroom activities** | 6,727 |  |  |  |  |  |  |  |  |  |
| Always/almost always |  | 5,174 (76.9) | 4,542 (79.1) | 553  (65.0) | 79  (58.5) |  | Ref |  |  |  |
| Sometimes |  | 1,406 (20.9) | 1,094 (19.1) | 263  (30.9) | 49  (36.3) |  | 1.97  (1.68-2.32) | 1.49  (1.24-1.79) | 2.58  (1.80-3.70) | 1.51  (0.95-2.41) |
| Hardly ever/never |  | 147  (2.2) | 105  (1.8) | 35  (4.1) | 7  (5.2) | <0.001^d^ | 2.76  (1.87-4.08) | 1.90  (1.20-3.00) | 3.85  (1.74-8.51) | 2.84  (1.13-7.11) |
| **High level of involvement in self-chosen activities** | 6,732 |  |  |  |  |  |  |  |  |  |
| Always/almost always |  | 5,071 (75.3) | 4,468 (77.8) | 535  (62.8) | 68  (50.4) |  | Ref |  |  |  |
| Sometimes |  | 1,476 (21.9) | 1,159 (20.2) | 263  (30.9) | 54  (40.0) |  | 1.90  (1.61-2.23) | 1.56  (1.30-1.87) | 3.05  (2.13-4.39) | 1.82  (1.14-2.90) |
| Hardly ever/never |  | 185 (2.7) | 118 (2.1) | 54  (6.3) | 13  (9.6) | <0.001^d^ | 3.81  (2.73-5.32) | 2.62  (1.78-3.86) | 6.92  (3.68-12.99) | 5.69  (2.73-11.84) |
| **Uses activities and resources independently** | 6,727 |  |  |  |  |  |  |  |  |  |
| Always/almost always |  | 4,135 (61.5) | 3,702 (64.5) | 394  (46.1) | 39  (28.7) |  |  |  |  |  |
| Sometimes |  | 2,254 (33.5) | 1,797 (31.3) | 379  (44.4) | 78  (57.4) |  | 1.98  (1.70-2.31) | 1.65  (1.39-1.96) | 4.14  (2.81-6.11) | 2.75  (1.71-4.40) |
| Hardly ever/never |  | 338  (5.0) | 238  (4.1) | 81  (9.5) | 19  (14.0) | <0.001^d^ | 3.20  (2.44-4.21) | 2.23  (1.63-3.07) | 7.51  (4.26-13.23) | 5.79  (2.91-11.52) |
| **Interested, motivated, and excited to learn** | 6,724 |  |  |  |  |  |  |  |  |  |
| Always/almost always |  | 4,379 (65.1) | 3,918 (68.3) | 406  (47.8) | 55  (40.4) |  |  |  |  |  |
| Sometimes |  | 2,000 (29.7) | 1,575 (27.4) | 364  (42.9) | 61  (44.9) |  | 2.23  (1.91-2.60) | 1.84  (1.55-2.19) | 2.75  (1.90-3.97) | 2.22  (1.39-3.54) |
| Hardly ever/never |  | 345 (5.1) | 246 (4.3) | 79  (9.3) | 20  (14.7) | <0.001^d^ | 3.11  (2.37-4.10) | 2.08  (1.51-2.87) | 5.78  (3.41-9.81) | 4.34  (2.19-8.59) |
| **Confident to try new things, initiate ideas and speak in familiar group** | 6,721 |  |  |  |  |  |  |  |  |  |
| Always/almost always |  | 4,028 (59.9) | 3,609 (62.9) | 363  (42.6) | 56  (42.1) |  | Ref |  | Ref |  |
| Sometimes |  | 2,193 (32.6) | 1,785 (31.1) | 363  (42.6) | 45  (33.8) |  | 2.02  (1.73-2.36) | 1.72  (1.45-2.04) | 1.64  (1.11-2.44) | 1.61  (0.99-2.61) |
| Hardly ever/never |  | 500 (7.4) | 342 (6.0) | 126  (14.8) | 32  (24.1) | <0.001^d^ | 3.66  (2.91-4.61) | 2.47  (1.89-3.23) | 6.13  (3.92-9.58) | 4.92  (2.78-8.69) |
| **Maintains attention and concentrates** | 6,719 |  |  |  |  |  |  |  |  |  |
| Always/almost always |  | 3,813 (56.7) | 3,426 (59.8) | 346  (40.6) | 41  (30.4) |  | Ref |  | Ref |  |
| Sometimes |  | 2,420 (36.0) | 1,950 (34.0) | 397  (46.6) | 73  (54.1) |  | 2.02  (1.73-2.36) | 1.75  (1.47-2.09) | 3.10  (2.12-4.55) | 1.75  (1.09-2.80) |
| Hardly ever/never |  | 487 (7.2) | 356 (6.2) | 109  (12.8) | 21  (15.6) | <0.001^d^ | 3.05  (2.39-3.88) | 2.30  (1.73-3.05) | 4.91  (2.87-8.39) | 3.35  (1.76-6.37) |
| **Perseveres when problem solving** | 6,714 |  |  |  |  |  |  |  |  |  |
| Always/almost always |  | 3,676 (54.8) | 3,324 (58.0) | 319  (37.5) | 33  (25.2) |  | Ref |  |  |  |
| Sometimes |  | 2,397 (35.7) | 1,958 (34.2) | 383  (45.0) | 56  (42.7) |  | 2.04  (1.88-4.48) | 1.78  (1.49-2.12) | 2.91  (1.88-4.48) | 2.14  (1.26-3.65) |
| Hardly ever/never |  | 641  (9.5) | 450  (7.9) | 149  (17.5) | 42  (32.1) | <0.001^d^ | 3.46  (2.79-4.31) | 2.43  (1.89-3.13) | 9.49  (5.95-15.13) | 6.60  (3.62-12.03) |

^a^ Typical cognitive development, ^b^ Below average cognitive ability, ^c^ Low cognitive ability, ^d^ Pearson’s Chi-squared test, ^e^ CI - Confidence Interval, ^f^ Adjusted for child gender, gestational age, siblings, primary caregiver education, household income, parent-child relationship, child BMI, cultural background, child temperament, partner in household, maternal mental health

Supplemental Table S7. Teacher-reported emotional behavioural function at age 9 years

|  | Valid | Total | TCD  (GCA >1SD below mean)  n=6,286 | BACA  (GCA 1-2 SD below mean)  n=952 | LCA  (GCA >2SD below mean)  n=154 | p-value | BACA^a^ v TCD^b^  Odds ratio  (95% CI^e^) | BACA v TCD  Adjusted odds ratio^f^ (95% CI^e^) | LCA^c^ v TCD  Odds ratio  (95% CI^e^) | LCA v TCD  Adjusted odds ratio^f^ (95% CI^e^) |
| --- | --- | --- | --- | --- | --- | --- | --- | --- | --- | --- |
| Total Difficulties | 6,739 |  |  |  |  |  |  |  |  |  |
| Average |  | 5,680 (84.3) | 4,923 (85.6) | 663 (77.6) | 94 (69.1) |  | Ref | Ref | Ref | Ref |
| High |  | 1,059  (15.7) | 826  (14.4) | 191  (22.4) | 42  (30.9) | <0.001^d^ | 1.71  (1.44-2.05) | 1.27  (1.03-1.56) | 2.64  (1.82-3.83) | 1.95  (1.20-3.17) |
| Emotional | 6,740 |  |  |  |  |  |  |  |  |  |
| Average |  | 5,632 (83.6) | 4,853 (84.4) | 675 (79.0) | 104 (76.5) |  | Ref | Ref | Ref | Ref |
| High |  | 1,108  (16.4) | 897  (15.6) | 179  (21.0) | 32  (23.5) | <0.001^d^ | 1.38  (1.14-1.67) | 1.16  (0.95-1.42) | 2.23  (1.50-3.33) | 1.89  (1.19-3.02) |
| Conduct | 6,740 |  |  |  |  |  |  |  |  |  |
| Average |  | 6,118 (90.8) | 5,242 (91.2) | 753 (88.2) | 13 (90.4) |  | Ref | Ref | Ref | Ref |
| High |  | 622  (9.2) | 508  (8.8) | 101  (11.8) | 13  (9.6) | 0.019^d^ | 1.38  (1.10-1.73) | 0.97  (0.74-1.26) | 1.08  (0.60-1.93) | 0.58  (0.27-1.24) |
| Hyperactivity | 6,739 |  |  |  |  |  |  |  |  |  |
| Average |  | 5,641 (83.7) | 4,895 (85.1) | 662 (77.5) | 84 (61.8) |  | Ref | Ref | Ref | Ref |
| High |  | 1,098  (16.3) | 854  (14.9) | 192  (22.5) | 52  (38.2) | <0.001^d^ | 1.66  (1.39-1.98) | 1.29  (1.05-1.59) | 3.52  (2.47-5.01) | 2.58  (1.60-4.15) |
| Peer problems | 6,739 |  |  |  |  |  |  |  |  |  |
| Average |  | 5,799 (86.1) | 5,000 (87.0) | 700 (82.0) | 99 (73.3) |  | Ref | Ref | Ref | Ref |
| High |  | 940  (13.9) | 750  (13.0) | 154  (18.0) | 36  (26.7) | <0.001^d^ | 1.46  (1.21-1.77) | 1.06  (0.85-1.32) | 2.42  (1.64-3.57) | 2.29  (1.44-3.64) |
| Prosocial | 6,737 |  |  |  |  |  |  |  |  |  |
| Average |  | 5,888 (87.4) | 5,062 (88.1) | 717 (84.2) | 109 (80.1) |  |  |  |  |  |
| High |  | 849  (12.6) | 687  (11.9) | 135  (15.8) | 27  (19.9) | <0.001^d^ | 1.39  (1.14-1.70) | 1.00  (0.79-1.26) | 1.83  (1.19-2.82) | 0.92  (0.52-1.63) |

^a^ Below average cognitive ability, ^b^ Typical cognitive development, ^c^ Low cognitive ability, ^d^ Pearson’s Chi-squared test, ^e^ CI - Confidence Interval, ^f^ Adjusted for child gender, gestational age, siblings, primary caregiver education, household income, parent-child relationship, child BMI, cultural background, child temperament, partner in household, maternal mental health

Supplemental Table S8. Child-reported experience of school at age 9 years

|  | Valid | Total | Typical cognitive development | Below average cognitive ability | p-value | Odds ratio  (95% CI^b^) | Adjusted odds ratio^c^ (95% CI) | p-value |
| --- | --- | --- | --- | --- | --- | --- | --- | --- |
| Thoughts about school | 7,304 |  |  |  |  |  |  |  |
| Always like it |  | 2,409 (33.3) | 2,088 (33.6) | 321 (29.5) |  | Ref | Ref |  |
| Sometimes like it |  | 4,494 (61.5) | 3,837 (61.7) | 657 (60.3) |  | 1.11 (0.97-1.29) | 1.01 (0.86-1.18) | 0.944 |
| Never like |  | 401 (5.5) | 290 (4.7) | 111 (10.2) | <0.001^a^ | 2.49 (1.95-3.20) | 1.82 (1.37-2.43) | <0.001 |
| Self-reported performance | 7,299 |  |  |  |  |  |  |  |
| Well |  | 4,014 (55.0) | 3,488 (56.2) | 526 (48.3) |  | Ref | Ref |  |
| Average |  | 3,195 (43.8) | 2,668 (43.0) | 527 (48.4) |  | 1.31 (1.15-1.49) | 1.37 (1.18-1.58) | <0.001 |
| Poorly |  | 90 (1.2) | 54 (0.9) | 36 (3.3) | <0.001^a^ | 4.39 (2.85-6.76) | 3.04 (1.85-4.98) | <0.001 |
| Reported being picked on | 6,978 |  |  |  |  |  |  |  |
| No |  | 4,111 (58.8) | 3,573 (59.9) | 538 (53.2) |  |  | Ref |  |
| Yes |  | 2,867 (41.1) | 2,394 (40.1) | 473 (46.8) | <0.001^a^ | 1.31 (1.15-1.50) | 1.27 (1.09-1.48) | 0.002 |
| Reported picking on someone | 6,872 |  |  |  |  |  |  |  |
| No |  | 5,819 (84.7) | 5,045 (86.0) | 774 (77.0) |  |  |  |  |
| Yes |  | 1,053 (15.3) | 822 (14.0) | 231 (23.0) | <0.001^a^ | 1.83 (1.55-2.16) | 1.53 (1.27-1.84) | <0.001 |

^a^ Pearson’s Chi-squared test, ^b^ CI - Confidence Interval, ^c^ Adjusted for child gender, gestational age, siblings, primary caregiver education, household income, parent-child relationship, child BMI, cultural background, child temperament, partner in household, maternal mental health

Supplemental Figure S1. Directed acyclic graph depicting the causal relationship between cognitive ability at age 5 and child-reported school-experience at age 9


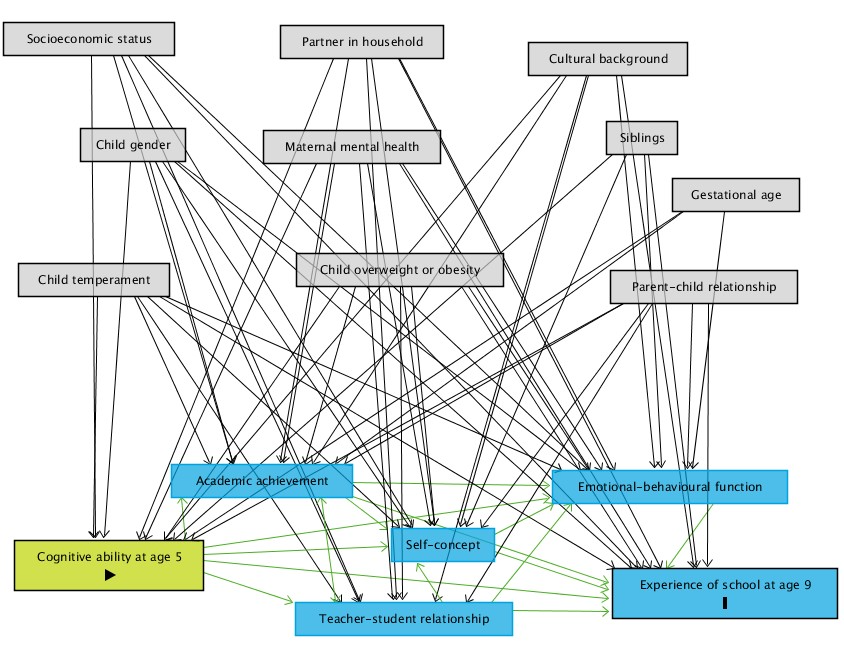


Figure S1 Legend

Exposure

Outcome

Ancestor of outcome

Adjusted variable

Causal pathway

Unobserved variable

For clarity of presentation, lines representing relationships between confounding variables have been removed.

**Estimand of interest:** The total causal effect of cognitive ability at age 5 on the child-reported experience of school at age 9.

**Minimally sufficient DAG implied adjustment set:** Socioeconomic status, partner in household, cultural background, child gender, maternal mental health, siblings, child temperament, child overweight or obesity, parent-child relationship

Supplemental Figure S2. Directed acyclic graph depicting the causal relationship between cognitive ability at age 5 and child’s self-concept at age 9


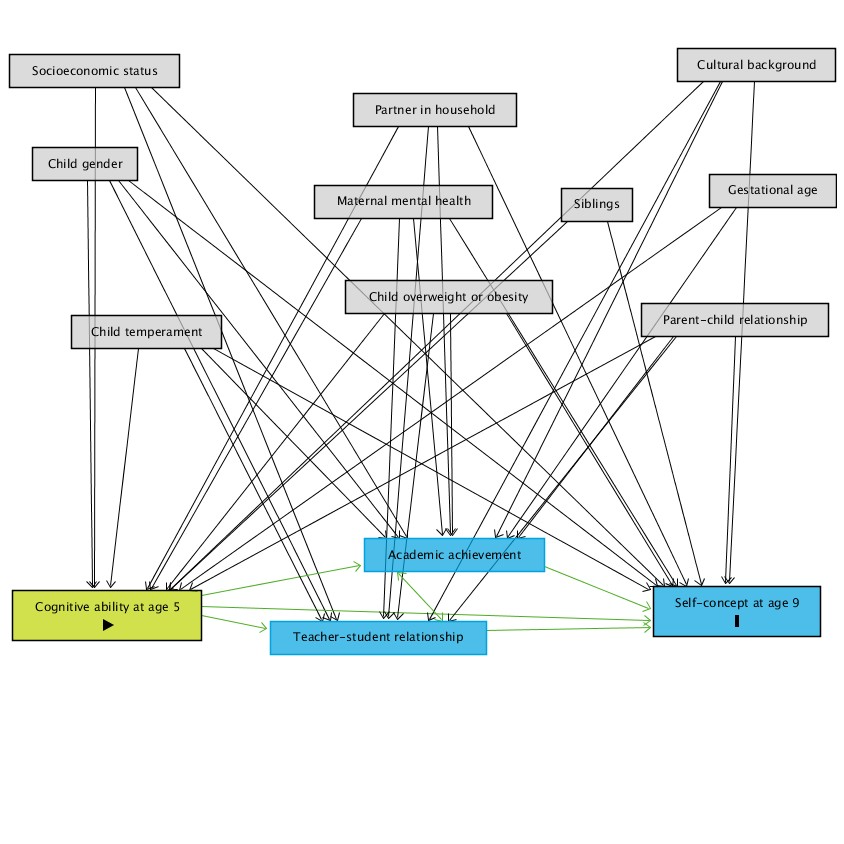


Figure S2 Legend

Exposure

Outcome

Ancestor of outcome

Adjusted variable

Causal pathway

For clarity of presentation, lines representing relationships between confounding variables have been removed.

**Estimand of interest:** The total causal effect of cognitive ability at age 5 on the child self-concept at age 9

**Minimally sufficient DAG implied adjustment set:** Socioeconomic status, partner in household, cultural background, child gender, maternal mental health, siblings, child temperament, child overweight or obesity, parent-child relationship

Supplemental Figure S3. Directed acyclic graph depicting the causal relationship between cognitive ability at age 5 and class engagement at age 9


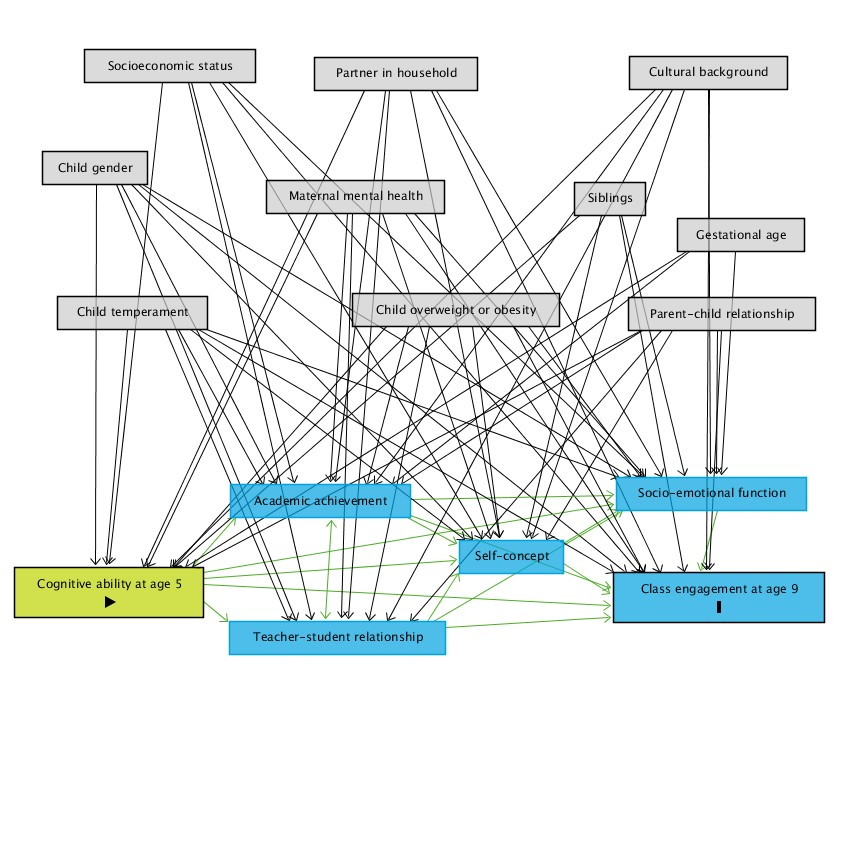


Figure S3 Legend

Exposure

Outcome

Ancestor of outcome

Adjusted variable

Causal pathway

For clarity of presentation, lines representing relationships between confounding variables have been removed.

**Estimand of interest:** The total causal effect of cognitive ability at age 5 on the teacher-reported class engagement at age 9

**Minimally sufficient DAG implied adjustment set:** Socioeconomic status, partner in household, cultural background, child gender, maternal mental health, siblings, child temperament, child overweight or obesity, parent-child relationship

Supplemental Figure S4. Directed acyclic graph depicting the causal relationship between cognitive ability at age 5 and emotional-behavioural function at age 9


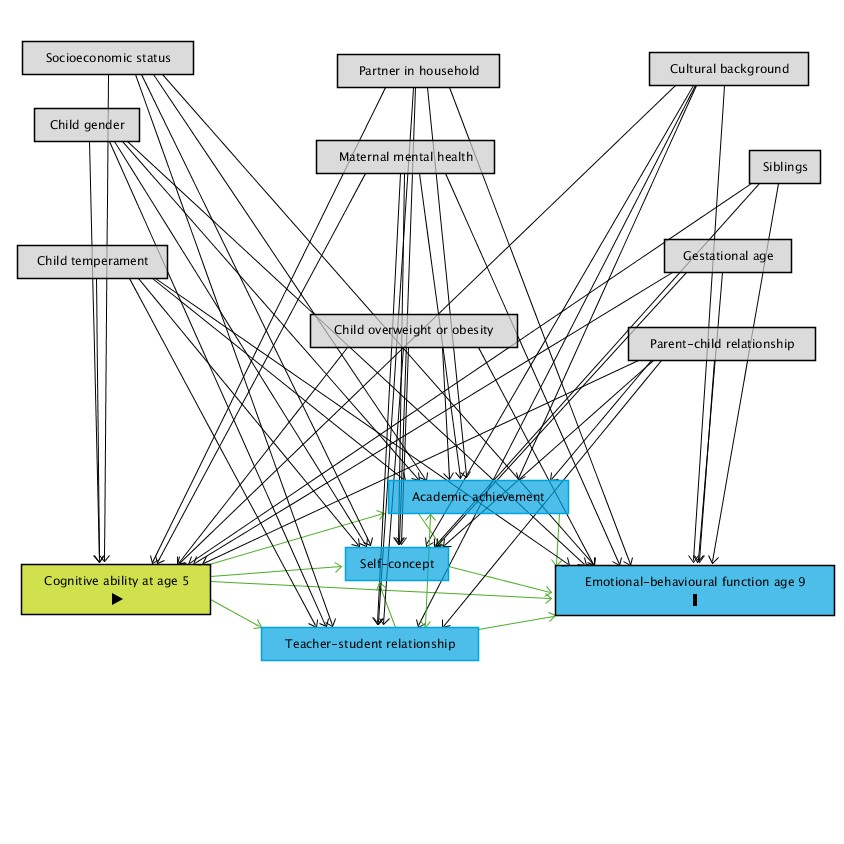


Figure S4 Legend

Exposure

Outcome

Ancestor of outcome

Adjusted variable

Causal pathway

For clarity of presentation, lines representing relationships between confounding variables have been removed.

**Estimand of interest:** The total causal effect of cognitive ability at age 5 on the teacher-reported emotional-behavioural function at age 9

**Minimally sufficient DAG implied adjustment set:** Socioeconomic status, partner in household, cultural background, child gender, maternal mental health, siblings, child temperament, child overweight or obesity, parent-child relationship
